# Assessment of paradoxical anterior translation in a CR total knee prosthesis coupling dynamic RSA and FE techniques

**DOI:** 10.1186/s40634-021-00361-y

**Published:** 2021-07-10

**Authors:** Agostino Igor Mirulla, Laura Bragonzoni, Stefano Zaffagnini, Tommaso Ingrassia, Raffaele Zinno, Bernardo Innocenti

**Affiliations:** 1 Department of Engineering, University of Palermo, Palermo, Italy; 2 Department of Biomedical and Neuromotor Sciencies, Università di Bologna, Bologna, BO Italy; 3 Department for Life Quality Studies, University of Bologna, Rimini, Italy; 4 2nd Orthopaedic and Traumatologic Clinic, IRCCS Istituto Ortopedico Rizzoli, Bologna, Italy; 5 BEAMS Department (Bio Electro and Mechanical Systems), Université Libre de Bruxelles, Bruxelles, Belgium

**Keywords:** Dynamic RSA, FE analysis, TKA, Mobile bearing, Kinematics

## Abstract

**Purpose:**

The study aims were to assess the kinematic data, Internal-External (IE) rotation, and Antero-Posterior (AP) translation of the contact points between the femoral condyles and polyethylene insert and to develop a combined dynamic RSA-FE (Radiostereometric – Finite Element) model that gives results congruent with the literature.

**Methods:**

A cohort of 15 patients who underwent cemented cruciate-retaining highly congruent mobile-bearing total knee arthroplasty were analyzed during a sit-to-stand motor task. The kinematical data from Dynamic RSA were used as input for a patient-specific FE model to calculate condylar contact points between the femoral component and polyethylene insert.

**Results:**

The femoral component showed an overall range about 4 mm of AP translation during the whole motor task, and the majority of the movement was after 40° of flexion. Concerning the IE rotation, the femoral component started from an externally rotate position (− 6.7 ± 10°) at 80° of flexion and performed an internal rotation during the entire motor task. The overall range of the IE rotation was 8.2°.

**Conclusions:**

During the sit to stand, a slight anterior translation from 40° to 0° of flexion of the femoral component with respect to polyethylene insert, which could represent a paradoxical anterior translation. Despite a paradoxical anterior femoral translation was detected, the implants were found to be stable. Dynamic RSA and FE combined technique could provide information about prosthetic component’s stress and strain distribution and the influence of the different designs during the movement.

## Background

Total knee arthroplasty (TKA) is the gold standard treatment for patients with primary osteoporosis, as it can relief pain and restore joint function. The prosthetic implant has a survival rate of 82% at 25 years [14]. Moreover, a patient satisfaction about 80–90% after 1-5 years has been reported [5, 10]. The main causes of TKA failure and revision are infection, aseptic loosening, periprosthetic fracture, stiffness, and instability [26, 28]. The last one may be evaluated through knee kinematics using different techniques, both in vitro and in vivo [2, 9].

Model-based RSA is already used to assess in vivo knee kinematics in several prosthesis designs during daily life motor tasks [1, 7, 21, 27]. Although dynamic RSA allows to analyze in-vivo knee kinematics, it does not provide with the same accuracy the exactly contact points between the femoral condyles and polyethylene insert. Furthermore, the kinematical data have been assessed frame by frame not considering the mechanical patterns of the knee prosthetic materials, such as deformation and surface contact behavior [9].

To reach this goal, Catani et al. [9] reported a technique using the in vivo 3D kinematics obtained from fluoroscopy as input for FE models in order to determinate the contact points between the femoral component and the insert in patients with TKA. This method was recently used by another study that combined the kinematic data carried out by dynamic fluoroscopy with FE models in order to investigate articular surface contacts, both at the condyles and at the post-cam [2].

As reported in the literature [4, 24], custom specific knee finite element models (FEM) has recently been developed, both in clinical applications and in the process of medical devices design [13, 23], to investigate native and replaced knee joint kinematics and kinetics [6, 15, 18]. These models represent a valid alternative to in-vivo or experimental assessments since they were able to provide results comparable to those two methods while maintaining lower cost in comparison [2, 9].

In this study, an innovative technique, combining dynamic RSA and patient-specific finite element models, was applied to analyze a group of 15 patients who underwent total knee arthroplasty with a cemented CR highly congruent MB TKA during the execution of a sit to stand from chair. Specifically, this technique utilized real in vivo 3D kinematics obtained from RSA dynamic as input data for finite element analyses of the prosthesis.

The aim of our study was to assess the kinematic data, Internal-External (IE) rotation and Antero-Posterior (AP) translation of the contact points between the femoral condyles and polyethylene insert, through a patient-specific FE model, based on a validated FE technique [9, 16]. The secondary purpose was to develop a combined dynamic RSA-FE model that give results congruent with the literature [7, 12, 29].

The hypothesis of the present study was that the kinematic data acquired by dynamic RSA may be used to evaluate the contact points translation at polyethylene-femoral component interface by finite element analysis obtaining results congruent with the literature.

## Materials and methods

The patient recruitment, demographic data, study method, and kinematic data analyzed through dynamic RSA using in this work have been acquired according to the Ethics approval by Institutional Review Board (IRB) of XXX Institute (IRCCS) (ID: 0035595 October 22,2015), and have been already published [7].

Briefly, Cardinale et al. [7] randomly selected a cohort of 15 patients who underwent cemented CR highly congruent MB TKA (Gemini, Waldemar LINK GmbH & Co. KG, Barkhausenweg 10, 22,339 Hamburg, Germany) with patella resurfacing for primary osteoarthritis (OA). The evaluation was performed after a minimum nine-month follow-up using Model-based dynamic RSA in weight-bearing conditions and during the execution of a sit to stand from chair. The RSA methods and accuracy are previously published [1, 7, 21, 27].

The validated dynamic RSA method allows to measure with sub-millimetric accuracy [3] (average 0.2 mm, SD ± 0.5 mm for the model position, and 0.3° ± 0.2° for the model orientation), according to the ISO − 5725 regulation [30].

**Fig. 1:**
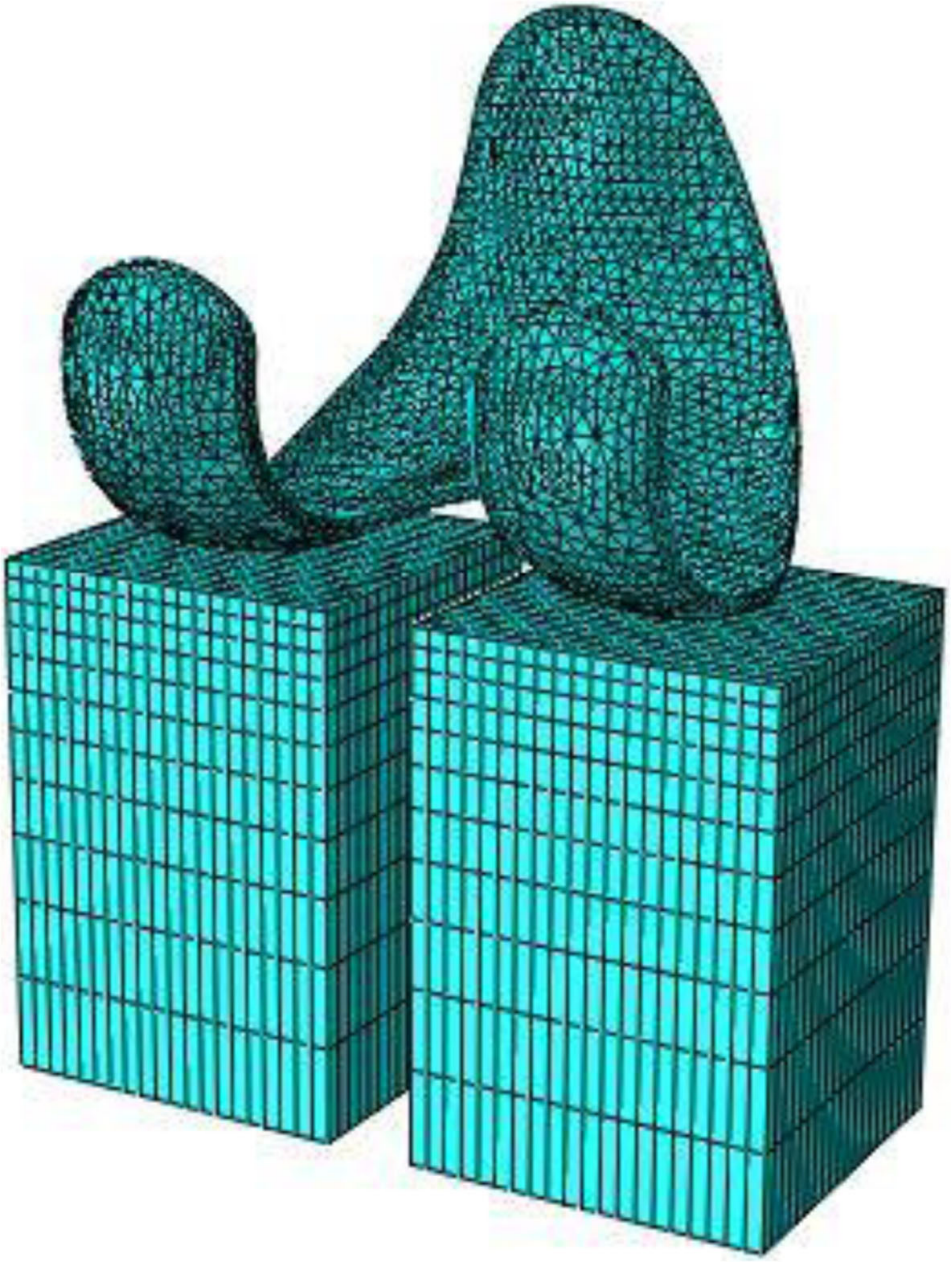
Mesh of femoral component and boxes

The kinematical data acquired thought Dynamic RSA were used as input for a patient-specific FE models, developed on the basis of a validated FE technique [9], to calculate condylar contact points between the femoral component and polyethylene insert. The first FE model of the femoral component and polyethylene insert was developed in Abaqus/Explicit version 2019 (Dassault Systèmes, Vélizy-Villacoublay, France) from the original CAD models of the implant provided by the manufacturer. Three size femoral components (CR2, CR3, CR5) were used and considered as a rigid surface and represented by triangular surface elements with 2 mm element size (*3233 elements for CR2, 3750 elements for CR3 and 4576 elements for CR5) (Fig. 1). The polyethylene insert was modelled by two fixed box and represented by eight-node 3D hexahedral elements. To create an element size variation from 2 mm (surface in contact with femoral component) to 10 mm, a single bias was applied in z-direction, obtaining 4400 elements for each box (Fig. 1).

Initially, the boxes were considered fixed, and the femoral component moved according to the in vivo relative kinematics obtained from RSA. Subsequently, to avoid excessive polyethylene deformation or lift-off between the femoral condyles and the insert, and to guarantee a constant penetration of 1 mm, the fixed boxes was replaced with movable boxes. Following previous model [2, 9], the latters were assumed to move in the superior-inferior direction together the femoral component using the superior-inferior displacement of the contact points calculated in the first analysis. No movement was allowed for the boxes in antero-posterior and medio-lateral direction.

Young’s modulus and Poisson ratio assumed for the femoral component, assumed linear elastic isotropic, were, respectively, 240 GPa and 0.3 [17]. The polyethylene insert was treated as a homogenous and isotropic material according to literature data [8, 16–19, 25].

The contact points were determined by the FE software as the centroid of the pressure distribution between the femoral condyles and the polyethylene insert [11].

**Fig. 2:**
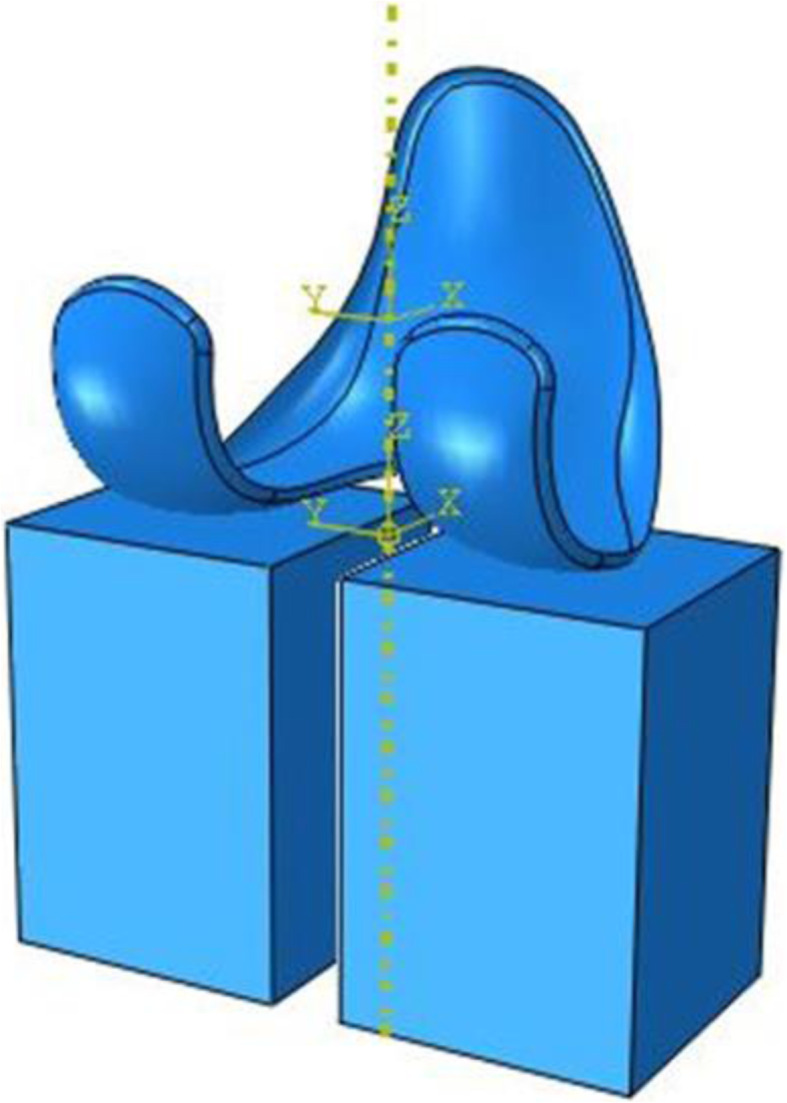
Initial relative position between the femoral component and the boxes

As output, for each patient, the IE rotation and the AP translation of the whole femoral component was calculated. Moreover, the AP displacement of the medial and lateral compartments, normalized with respect to prosthesis size, were evaluated and all kinematical results were plotted versus the knee flexion angle. All reported data, calculated for each patient, were evaluated starting from the same initial position, aligning the coordinate systems of femoral component and of polyethylene on z-axis (Fig. 2).

## Results

**Fig. 3:**
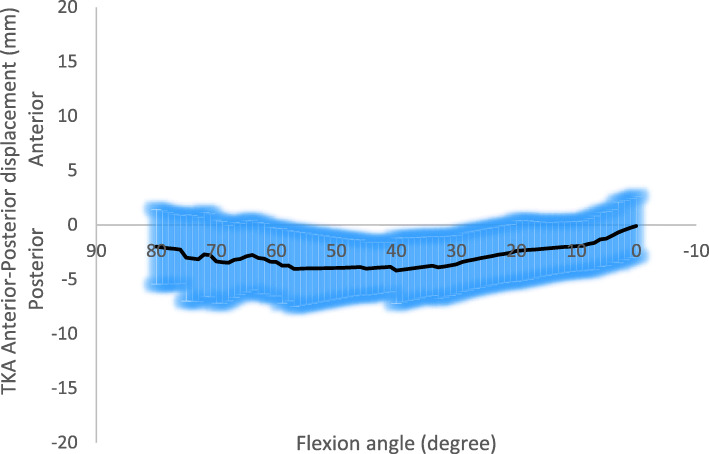
Total knee arthroplasty Anterior-Posterior translation (mean and standard deviation)

The AP translation (Fig. 3) showed that the femoral component started from a posterior position (− 2 mm ± 3.4 mm) at 80° of flexion, kept an almost constant position up to 40° and translate anteriorly during the last 40° of flexion. In average, the AP translation overall range during the whole motor task was about 4 mm.

**Fig. 4:**
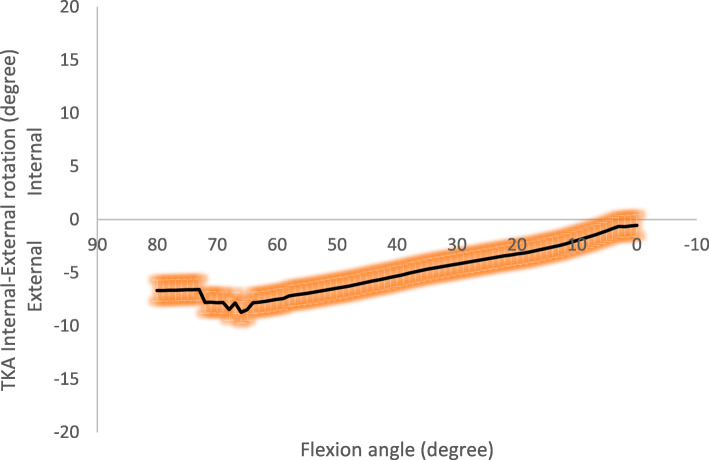
Total knee arthroplasty Internal-External rotation (mean and standard deviation)

Analysing the IE rotation plotted versus knee flexion, the femoral component started from an externally rotate position (− 6.7 **±** 10°) at 80° of flexion and performed an internal rotation during the entire motor task (Fig. 4). The overall range of the IE rotation was 8.2°.

**Fig. 5:**
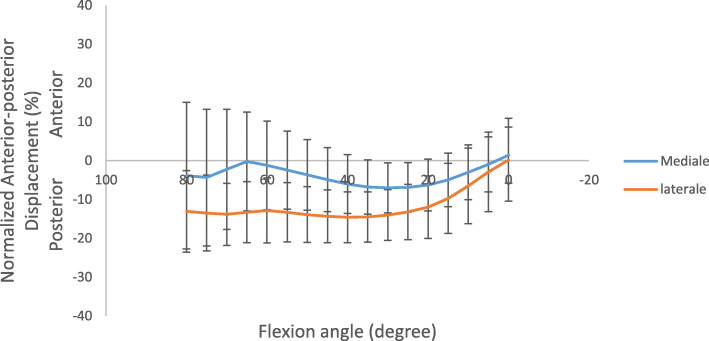
Normalized Anterior-Posterior translation of the medial and lateral condyles

**Fig. 6:**
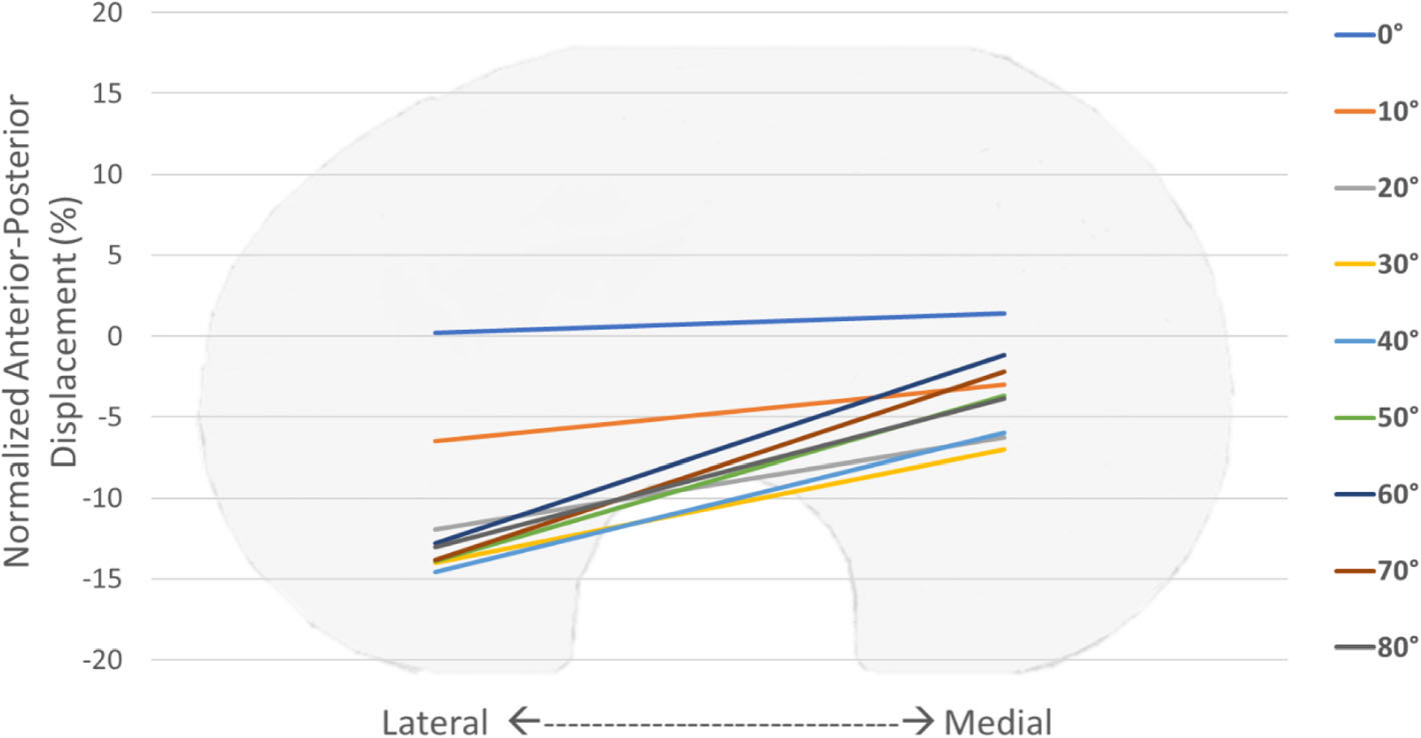
Normalized Anterior-Posterior translation of the femoral compartment

The normalized AP translation, reported as percentage value of the length of the prosthesis (Fig. 5), showed that both medial and lateral condyles started from posterior position and moved in anterior direction during the motor task. An almost constant offset of about 8% was observed between 80° and 40° of flexion. Later, the offset decreased between 40° and 0° of flexion (from 8% to 1%) bringing the AP position of both condyles almost at the same position in full extension. Analysing the overall AP translation and IE rotation ranges (Fig. 6), a greater motion in AP direction occurred in low flexion angle then in high ones. Whilst, the femoral component maintained an extra-rotated position from 80° to 40°, and performed an internal-rotation in the last degrees of flexion.

## Discussion

The most important finding of the present study was that during the sit-to-stand, a slight anterior translation was observed from 40° to 0° of flexion in the femoral component with respect to polyethylene insert, which could represent a paradoxical anterior translation as reported in several studies [7, 12, 29].

The dynamic RSA technique allows to accurately investigate in-vivo the kinematical behavior of a total knee prosthesis analyzing the relative movement between femoral compartment and tibial plateau frame by frame. Instead, the FE analysis provides more information taking account both kinematical, kinetic and biomechanical behaviors. The innovative feature of the present study is the combination of dynamic RSA technique and finite element analysis. The reliability of a combined technique, finite element analysis and in vivo 3D fluoroscopic kinematics, was already tested and confirmed [2, 9].

The paradoxical anterior translation is an important and very common result that could be related to different factors and may cause implant instability. Analyzing the literature, higher AP translation could be led by a not congruent prosthesis design and by cruciate resection [20].

Based on these considerations, the slight AP range reported in this study could be associated to the high congruent design and to the anterior cruciate retaining, typical of the CR mobile bearing TKA [16].

Analyzing the normalized AP translation reported in Fig. 6, the femoral component performed a medial pivot movement during the whole task starting from an external rotation, coherently with the results reported in the literature [22].

As showed by results, a similar kinematical behavior was observed in all patients. Despite a paradoxical anterior femoral translation was detected, the implants showed a great stability. It could be related to the highly congruent polyethylene design.

Dynamic RSA and FE combined technique could provide information about prosthetic component’s stress and strain distribution and the influence of the different designs during the movement.

This study presents some limitations. Firstly, the number of patients is not enough large to produce strong evidence, although it is in line with similar studies [2, 7]. Secondly, all patients performed the sit-to stand motor task without standardisation not providing homogeneous data, but ensuring the most natural movement as possible. Finally, this study investigated only the extension phase of movement, not finding a good comparison with the literature where most of the studies that assessed in vivo kinematical patterns were focused on flexion movement.

## Conclusion

During the sit-to-stand, a paradoxical anterior translation was detected, according to the literature. Dynamic RSA and FE combined technique could provide information about prosthetic component’s stress and strain distribution and the influence of the different designs during the movement.

An important outcome that should be analyzed is the success of the TKA during long time period and the influence of the mechanical behavior. For this reason, future studies will be focused on long term follow up and on other motor tasks.

## Data Availability

The datasets used and/or analyzed during the current study are available from the corresponding author on reasonable request.

## Abbreviations

PCL: Posterior cruciate retaining
OA: Osteoarthritis
CR: Cruciate retaining
MB: Mobile bearing
TKA: Total knee arthroplasty
RSA: Roentgen stereophotogrammetric analysis
FE: Finite element
FEM: Finite element method
IE: Internal-external
AP: Anterior-Posterior
